# Engaging adults in organized physical activity: a scoping review of recruitment strategies

**DOI:** 10.1093/heapro/daad050

**Published:** 2023-05-26

**Authors:** Ruth Mackenzie-Stewart, Cassandra de Lacy-Vawdon, Niamh Murphy, Ben J Smith

**Affiliations:** Department of Public Health, School of Psychology and Public Health, La Trobe University, Melbourne, 3086, Australia; School of Public Health and Preventive Medicine, Monash University, Melbourne, 3800, Australia; Department of Public Health, School of Psychology and Public Health, La Trobe University, Melbourne, 3086, Australia; School of Public Health and Preventive Medicine, Monash University, Melbourne, 3800, Australia; Centre for Health Behaviour Research, Department of Sport and Exercise Science, South East Technological University, Waterford, Ireland; School of Public Health, University of Sydney, Sydney, 2006, Australia

**Keywords:** engagement, evaluation, health behavior, participation, physical activity

## Abstract

Scaling up established physical activity (PA) opportunities for broader population reach requires practitioners to carefully consider strategies implemented to recruit and attract new participants to their PA programs. This scoping review examines the effectiveness of recruitment strategies for engaging adults in organized (established and sustained) PA programs. Electronic databases were searched for articles published between March 1995 and September 2022. Qualitative, quantitative and mixed methods papers were included. Recruitment strategies were assessed against Foster *et al.* (Recruiting participants to walking intervention studies: a systematic review. *Int J Behav Nutr Phys Act* 2011;**8**:137–137.) assessment of quality for reporting recruitment and the determinants of recruitment rates were examined. 8394 titles and abstracts were screened; 22 articles were assessed for eligibility; 9 papers were included. Three of the 6 quantitative papers adopted a combination of passive and active recruitment strategies and 3 relied solely on active strategies. All 6 quantitative papers reported on recruitment rates; 2 evaluated the efficacy of recruitment strategies based on the achieved levels of participation. The evaluation evidence on how individuals are successfully recruited into organized PA programs, and how recruitment strategies influence or address inequities in PA participation, is limited. Culturally sensitive, gender sensitive and socially inclusive recruitment strategies based on building personal relationships show promise for engaging hard-to-reach populations. Improving the reporting and measurement of recruitment strategies into PA programs is essential to better understand which strategies are attracting various population groups thus allowing program implementers to employ recruitment strategies best suited to the needs of their community while making efficient use of program funding.

## BACKGROUND

Physical inactivity is a major risk factor for chronic diseases including heart disease, stroke, diabetes, cancers of the colon and breast, osteoporosis and depression (Bull *et al*., 2004; Bellew *et al.*, 2020). In Australia 47% of adults have at least one chronic health condition (Australian Bureau of Statistics [ABS], 2018). In 2016 the global age-standardized prevalence of physical inactivity for adults (18 years or older) was reported to be 27.5% (23.4 men; 31.7 women) (Guthold *et al*., 2018). In Australia the 2017–2018 National Health Survey found among adults aged 18–64 years 22.4% were insufficiently active [participating in any Physical activity (PA) less than 150 min in the previous week], and a further 11.5% were inactive (participating in 0 min of any PA in the previous week) (ABS, 2018). PA participation benefits extend to individuals, communities, and populations beyond chronic disease prevention. Evidence suggests a positive relationship between participation in organized PA demonstrates societal benefits such as feelings of social connectedness and happiness (Pels and Kleinert, 2016; Bellew, 2020; Budzynski-Seymour *et al.*, 2020; Sebastião and Mirda, 2021; Almevall *et al.*, 2022).

A high prevalence of physical inactivity places a substantial proportion of the population at increased risk of chronic disease (ABS, 2018); however, it is modifiable and organized PA programs provide one of many avenues for adults to increase their PA. Organized PA programs refers to sports or recreation activities organized by a club or association. Clubs and associations are not limited to sports organizations. Alternative organizations may include social clubs, church groups, old scholars’ associations, or gymnasiums (ABS, 2007). Across communities in developed countries there are a wide range of organized PA programs offering opportunities in both structured and semi-structured environments. These programs range from organized walking groups, circuit training, gyms and recreational PA programs through to social, structured and competitive PA program. Given the wide array of opportunities available a critical question for policy makers and practitioners working across the health, sport, recreation, and community services sectors is how to reach and engage individuals in various forms of organized PA.

Numerous studies have reported on recruitment into PA interventions within intervention trials, pilot programs or other forms of research (Rowland *et al.*, 2004; Jancey *et al.*, 2006; UyBico *et al.*, 2007; Mutrie *et al.*, 2010; Foster *et al.*, 2011; Cooke and Jones, 2017; Jong *et al.*, 2020), however, limited attention is paid to recruitment strategies for increasing attendance into established organized PA programs. This presents major challenges for improving implementation and scale-up of evidence from PA intervention trials and pilot programs particularly when considering the need to reduce disparities in participation among disadvantaged and hard-to-reach populations (Reis *et al.*, 2016). Optimizing effectiveness and population impact requires an understanding of strategies for engaging inactive participants into PA programs routinely delivered (Glasgow *et al.*, 1999; Froelicher and Lorig, 2002).

Recruitment is widely recognized as fundamental to program delivery influencing individual and population health outcomes including social inclusion and wellbeing (Lindsay Smith *et al.*, 2017). Recruitment of inactive participants into organized PA programs can be ongoing, costly and time-consuming for organizations (Peck *et al.*, 2008). In the context of PA recruitment has been defined as: ‘the process of inviting participation to a formal PA program, intervention, project, or event. This includes the invitation, informing and facilitation of interested adult participants to take part’ (Matthews *et al.*, 2012). Recruitment strategies can be considered active or passive and categorized on a spectrum from universal to purposive (Foster *et al.*, 2011).

To address this gap in evidence and determine priorities for future research this review seeks to address the following questions: What recruitment strategies have been reported to promote uptake of ongoing organized PA programs? What are the methods and findings of evaluations of recruitment strategies to enlist adults into ongoing organized PA programs?

This review intends to provide health promotion policy makers and practitioners with insight into the status of knowledge concerning recruitment for ongoing organized PA programs and to identify where efforts need to be focussed for increasing PA uptake.

## METHODS

This review identified the nature and extent of existing research and the limitations of the efficacy and determinants of recruitment strategies (Arksey and O’Malley, 2005). This review was undertaken using the Preferred Reporting Items for Systematic reviews and Meta-Analyses extension for Scoping reviews (PRISMA-ScR)(Tricco *et al.*, 2018). The scoping review questions and methodology, allowed for the inclusion of quantitative, qualitative, and mixed methods papers (Arksey and O’Malley, 2005; Peters *et al.*, 2021). The outcomes under review are recruitment rates and/or attendance into organized PA programs and the determinants of recruitment rates.

### Identification of papers/information sources

Electronic databases—Ovid Medline, CINAHL Plus, Informit Health Collection, Proquest Health, Psych-Info, Scopus, Cochrane library, Business source ultimate (previously known as Business source complete), SPORTDiscus, Communication and Mass Media Complete, Academic Search Complete, SocINDEX—were searched for peer reviewed papers published between March 1995 and September 2022. To complement the database searches, hand searching and snowballing of citations and references was completed by Authors 1 and 2. The search terms used were divided into three concepts, PA, recruitment strategies and participation, Supplementary file 1 contains the search terms for each concept.

### Eligibility

Articles are eligible where:

1. The PA programs and/or recruitment strategies were aimed at adults over the age of 18;
2. The PA programs were delivered in high-income countries;
3. Data on recruitment processes were reported, such as: source of recruitment for program attenders and/or non-attenders, reach of recruitment strategies, recruitment rates by behavioural theory stages and/or self-efficacy, recruitment rates by socio-demographic variables, experiences of recruitment strategies, barriers or facilitators for recruitment strategies, cost effectiveness of recruitment strategies;
4. The paper adopted a qualitative, quantitative, or mixed methods study design; and
5. The paper was peer reviewed, published in English between March 1995 and 2022.

Review papers and those reporting on recruitment into time limited pilots and intervention trials and were not maintained post the pilot or intervention period were excluded. Author 1 screened titles, abstracts, and full text articles; Author 2 confirmed inclusion of articles by screening the full text of each article. Authors 1, 2 and 4 agreed on all articles included in this review.

### Data extraction

Authors 1 and 4 developed the data extraction tables. For the purposes of data extraction and categorization of recruitment strategies and approaches this review has used revised definitions of active and passive recruitment (Foster *et al.*, 2011). See Supplementary file 3.

In addition to extracting whether the recruitment strategies adopted were passive and/or active, Author 2 extracted the following from each of the included articles: study design, population characteristics and size, location of program, recruitment methods, recruitment measures, qualitative variables examined, and recruitment results. Data extracted were reviewed for accuracy by Authors 1 and 4.

The following definitions were adopted when examining the potential audience for a recruitment strategy:

Random: Everyone in the study/intervention population has an equal chance of being exposed to the recruitment strategy, examples include newspaper articles, local and state-wide publications.

Universal: Everyone, including those not in the study population have equal chance of being exposed to the recruitment strategy, examples include public TV including, TV announcements, public service announcements and news coverage.

Convenience: Those in the target population will only be exposed to the recruitment strategy if they are conveniently located to accessible to the recruiter, examples include face to face recruitment at a train station or public place.

Purposive: The recruiters have defined the target population and employed recruitment strategies specifically to reach the defined population, for example, a GP identifying a patient meeting a specific population profile and is invited to participate.

Not Specified: Article does not specify the recruitment strategy used.

### Quality assessment

The Assessment of Recruitment Reporting Quality Scale (ARRQS) was used to record and rate the reporting of recruitment methods and outcomes in the included studies. The AARQS is a 5-point scale developed in 2011 (Foster *et al.*, 2011) with adaptions made in 2017 (Cooke and Jones, 2017). See Supplementary file 2. Each paper was scored independently by Authors 1 and 2. Scores of the quality of recruitment reporting in each paper are provided in Table 1.

**Table 1:** Characteristics of studies included in scoping review

	Citation	Study design	Study participants, location and size	Recruitment strategies	Active or passive recruitment strategy	Recruitment outcome measures and data collection	Variables reported in recruitment analysis	Results	Fosters *et al.* (2011) Recruitment reporting quality score
Quantitative studies	Bull and Milton (2010)	Process evaluation—quantitative descriptive statistics	Moderately active, moderately inactive and inactive patients from 6 general practices in London (*n* = 526)	Three study sites adopted **convenience** recruitment strategies using opportunistic recruitment by GP staff (in person) Three study sites used **purposive** or targeted recruitment from disease register (invitation letter).	Active	Recruitment rates by recruitment type Recruitment data tracked in Medical Information Systems	Age, gender, ethnicity	Opportunistic recruitment: 6% of consultations Targeted recruitment: 9%, 12% and 59% across 3 clinics Mean age: 54 years Screening by gender: 57% females and 43% males Screening by ethnicity: 54% Asian or Asian British, 19% white, 4% Black or Black British, mixed 1%, other 1%, missing 24%	4
	Chang *et al.* (2009)	Randomized Controlled Trial	Low-income, overweight or obese mothers aged 18-34 from a nutrition program in southern Michigan (*n* = 129)	**Purposive** recruitment strategies were adopted with participants invited in person by trained recruiters during a nutrition program. Cash incentives offered for participation	Active	Recruitment rates with comparison of participants and non-participants Self-administered demographics questionnaire and telephone interview administered survey	Age, education, smoking status, racial groups, BMI category	Reached 1007 women, 342 women (33.96%) completed screening, 194 (56.7%) were eligible. Of the 194 eligible, 129 (66.5%) were enrolled Compared to non-participants, enrolled participants were more likely to have some college education (*p* < 0.05); an average of 1.5 years older (*p* < 0.05); and, more likely to be former smokers (*p* < 0.05). No differences between racial groups or BMI categories	4
	Clarke and Eves (1997)	Cross-sectional Study	Middle-aged sedentary adults referred by their GP’s in the UK (*n* = 393)	A **convenience** recruitment strategy was adopted using opportunistic in person referrals from GP	Active	Recruitment rates by stage of readiness on the transtheoretical model GP administered survey with scales including: abbreviated 3 item version of stages of change measure, abbreviated 10 item version of the decisional balance measure, five item self-efficacy for exercise scale, and a 14 item composite Sport and Exercise Barriers Questionnaire	Stage of readiness age, gender, marital status, education level, home ownership, smoker status, alcohol use, medication use, exercise regularity.	673 participants referred, 393 (58.4%) volunteered to participate Participants classified into pre-contemplation (29, 7.4%), contemplation (189, 48.5%), and preparation stages (173, 44.1 %). Newman-Keuls follow-up analysis indicated all stages significantly different (*p* < 0.05) Males were significantly (*p* < 0.05) more confident than females to complete in exercise prescription (males, *M* = 5.98, SD = 1.39; females, *M* = 5.66, SD = 1.44)	3
	Coleman *et al.* (1997)	Randomized Controlled Trial	African American adults aged 55 years and older recruited from senior centres and the community in Seattle (*n* = 120)	**Purposive** recruitment strategies (phonathons) were supplemented with the use of **convenience** strategies (word of mouth, presentations, and classes), **random** strategies (print materials) and **universal** strategies (public TV)	Active and passive	Recruitment rates by method of recruitment Data were collected from recruitment activity logs, telephone screening questionnaires and baseline questionnaires and self-administered demographics questionnaire	Age, education level, ethnicity, marital status, gender, income level.	46% of total respondents became participants Participants recruited from phonathons (33%, *n* = 40), printed materials (33%, *n* = 39), word of mouth (26%, *n* = 31), and others (8%, *n* = 10) Mean age: 72 years Ethnicity: 77% African American, 22% Caucasian Gender: 80% female, 20% male Income level: 25% under $10 000, 24% over $25 000	4
	Kelly *et al.* (2019)	Process evaluation-quantitative descriptive statistics	Inactive adult men aged 18 + from eight counties in Ireland	A mix of recruitment strategies were adopted, including **purposive** recruitment strategies (text and email invitations, GP referrals); **convenience** strategies (word of mouth), **random** strategies (print materials) and **universal** strategies (local print, radio and social media campaigns)	Active and passive	Recruitment rates by method of recruitment Data were collected from self- administered baseline questionnaires	Age, ethnicity, educational attainment, relationship housing and employment status Self-reported PA participation, smoking, consumption of fruit, vegetables, and alcohol	927 men were recruited across all recruitment strategies including word of mouth (31.2%); newspaper or social media (23.3%); local service club (16.2%) local sports partnership (10.3%), family (8.4%), health services (5.8%) Across recruitment strategies adopted the Mean age was 50.7 years, 97.7% of those recruited were Irish, 42.7% had some or completed secondary school education. The majority were Married/cohabitating (77.6%) and living with family/wife/partner (85.2%) 84% of those recruited were not achieving 30 min of PA on at least 5 days per week	3
	Spencer *et al.* (1998)	Process evaluation—quantitative descriptive statistics	Inactive older adults aged 55–75 with osteoarthritis living in Washington state (*n* = 249)	A mix of recruitment strategies were adopted, including **purposive** recruitment strategies (letters sent from Arthritis Foundation and referrals from physicians); **convenience** strategies (Arthritis Foundation newsletters) and **universal** strategies (media and news coverage, advertising in local papers)	Active and passive	Recruitment rates by recruitment strategy and the associated cost of per participant recruited Data were collected using an eligibility screening tool and administrative information	Recruitment strategies compared by screened and enrolled responders for each strategy.	1018 people responded, 383 were eligible and 249 (65%) enrolled Gender: approximately 90% female Recruitment success rates: recruitment letters (30.5%), TV announcements (16.1%), public service announcements (16.1%), newspaper articles (9.6%), doctor referrals (12.1%), word of mouth (5.6%), ads (3%), flyers (3%) and others (1%) Expenses per participant: letters ($47), TV coverage ($0), public service announcements ($0), newspaper articles ($84), doctor referrals ($6), word of mouth ($0), and flyers ($42) No significant difference between recruitment methods of screened or enrolled respondents	4
Qualitative studies	Matthews *et al.* (2012)	Qualitative semi-structured interviews	Community-based walking program coordinators in the UK (*n* = 28)	Study participants were recruited **purposively** through organizations and referred by managers. Eligible coordinators contacted by phone and email Recruitment strategies as described by program delivers and coordinators	Active	Interviews analysed purpose and structure of walking groups, participant target groups, recruitment method selection, successes and failures, participant retention, and recruitment evaluation. Case studies used to appraise routine and culture Data were collected using semi-structured interviews	None	Recruited 28 of 37 walking coordinators identified Recruitment strategies with walking aims recruited (active) participants. Targeted methods with health aims recruited more specific (inactive) participants Only 5 of 28 groups used conceptual frameworks for recruitment; others recruited ad hoc Even spread of active (approaching people) and passive (waiting to be approached) recruitment methods. Word of mouth believed to be most effective method Marketing social benefits of walking more beneficial for recruitment than marketing health benefits	NA
	McCann *et al.* (2013)	Qualitative semi-structured interviews	Community health organizations delivering PA and health eating programs in Melbourne. 22 organizations agreed to participate, *n* = 25 semi-structured interviews undertaken.	Study participants were recruited **purposively** via organizations that delivered local community health programs focussed on PA and healthy eating Recruitment and retention strategies as described by those whom have delivered PA programs	Active	Semi-structured interviews provided summaries of recruitment and retention strategies used by program delivers. Focussing on describing who was recruited, how they were recruited and what programs they were recruited into and where the programs were delivered	None	Eight recruitment strategies emerged from the data set (in order or frequency): Word of mouth, links with key organizations and groups, printed materials, media, referrals, cross promotion of programs, and face to face Two recruitment and retention strategies were evident in the data set (in order of frequency): participant fees and support	NA
	Quirk and Haake (2019)	Qualitative semi-structured interviews	parkrun Long Term Health Conditions (LTHC) Outreach Ambassadors (OA) involved in PROVE for parkrun in England	Volunteer OA used **purposive** word of mouth, networking and personal engagement approaches to engaging with the community and those with LTHC while supporting parkrun volunteer communities with the purpose of encouraging and facilitating participation in parkrun events for those with LTHC	Active	Semi-structured telephones interviews and thematic analysis explored the experiences, support, success, skills and qualities, challenges of OA for increasing recruitment and engagement of those with LTHC	None	13 OA completed a semi-structured interview Thematic analysis showed that while parkrun lacked formalized supports, policies, and structures to engage with those with LTHC OA were able to engage with and recruit those with LTHC not already involved in parkrun The success of the OA recruitment strategy was driven by individual personal qualities, notably communication skills and experience with the LTHC Each LTHC group faces different challenges and barriers to participation ion parkrun and OA needed to be prepared to support addressing these barriers at the local event level. Due to the nature of parkrun, there are difficulties in demonstrating impact of the OA’s Historically, parkrun has relied on word of mouth as it’s only recruitment strategy, the use of OA’s represents an evolution in parkrun’s attempts to be inclusive of all community members	NA

### Compilation of results

Data extraction and charting data was used to produce a narrative account of the following characteristics: Context and population groups; study designs; recruitment approaches; recruitment measures; other measures; recruitment outcomes; qualitative studies; and a quality assessment of recruitment reporting.

## RESULTS

**Fig. 1:**
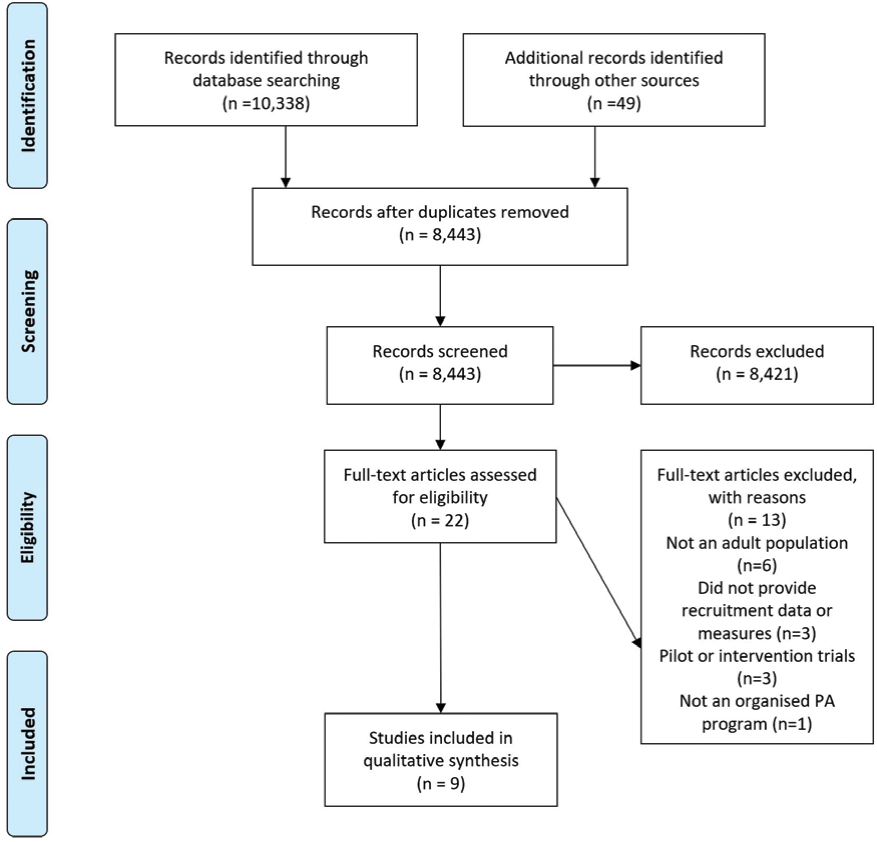
Flow diagram of selection process for articles included in review..

Searching yielded 10 387 citations following removal of duplicates (1944) 8443 were screened, resulting in 22 articles assessed for eligibility. Thirteen were excluded (reasons included: PA intervention pilots, not an organized PA program, did not provide recruitment data or measures, and not an adult population), resulting in 9 papers for inclusion (Clarke and Eves, 1997; Coleman *et al.*, 1997; Spencer *et al.*, 1998; Chang *et al.*, 2009; Bull and Milton, 2010; Matthews *et al.*, 2012; McCann *et al.*, 2013; Kelly *et al.*, 2019; Quirk and Haake, 2019), See Figure 1.

Papers originated from the USA (*n* = 3) (Coleman *et al.*, 1997; Spencer *et al.*, 1998; Chang *et al.*, 2009), the UK (*n* = 5) (Clarke and Eves, 1997; Bull and Milton, 2010; Matthews *et al.*, 2012; Kelly *et al.*, 2019; Quirk and Haake, 2019), and Australia (*n* = 1) (McCann *et al.*, 2013). Most participants in the papers included were female, ranging from 57 to 100%; 1 paper explicitly excluded females focussing on inactive males (Kelly *et al.*, 2019), 2 papers did not report the sex of participants (Matthews *et al.,* 2012; Quirk and Haake, 2019).

As shown in Tables 1 and 2 quantitative papers reported on recruitment into PA prescription programs in primary care settings (Clarke and Eves, 1997; Bull and Milton, 2010), 1 on recruitment into a diet, PA and stress management program (Chang *et al.*, 2009), 1 on a program conducted at a seniors centre (Coleman *et al.*, 1997), 1 into an aquatic exercise program (Spencer *et al.,* 1998), and 1 into a male only community-based PA program (Kelly *et al.*, 2019). Matthews *et al.* (Matthews *et al.*, 2012) and McCann *et al.* (McCann *et al.*, 2013) examined practices and perceptions of recruitment into organized walking groups and a PA and healthy eating program from the viewpoint of program staff, while Quirk and Haake (Quirk and Haake, 2019) examined Outreach Ambassadors (OA) as a recruitment strategy into parkrun, organized mass PA events.

**Table 2:** Summary of recruitment strategies and approach

Citation number	Active strategies	Passive strategies	Universal	Convenience	Purposive	Random
Bull and Milton (2010)	Opportunistic GP staff			X		
	Letters from disease register—targeted				X	
Chang *et al.* (2009)	Invitation from trained recruiters during nutrition program				X	
Clarke and Eves (1997)	Referral from GP			X		
Coleman *et al.* (1997)	Phonathons					X
	Word of mouth			X		
	Classes			X		
	Presentations at targeted sites				X	
		Print materials: flyers, brochures and posters		X		
		Public TV including TV announcements, public service announcements and news coverage	X			
Kelly *et al.* (2019)	In person text and email invitations via exiting databases				X	
	GP referrals			X		
	Word of mouth			X		
		Print materials	X			
		Local print, radio and social media campaigns	X			
Spencer *et al.* (1998)	Letters sent from Arthritis Foundation				X	
	Referrals from physicians			X		
		Arthritis Foundation newsletters		X		
		Media and news coverage, advertising in local papers	X			

The qualitative papers included in this review did not directly recruit participants into a PA program, therefore, are excluded from this table.

### Study designs

Six of the 9 papers adopted quantitative study designs (Clarke and Eves, 1997; Coleman *et al.*, 1997; Spencer *et al.*, 1998; Chang *et al.*, 2009; Bull and Milton, 2010; Kelly *et al.*, 2019). Spencer *et al.* (Spencer *et al.*, 1998), Bull and Milton (Bull and Milton, 2010), and Kelly *et al.* (Kelly *et al.*, 2019), presented results from post only process evaluations using descriptive statistics. Two used randomized controlled trial (RCT) designs (Clarke and Eves, 1997; Chang *et al.*, 2009) of an organized PA program, the interventions participants were recruited into have reportedly been sustained beyond the pilot and trial stages. One paper utilized a cross-sectional design (Clarke and Eves, 1997). Three included papers utilized qualitative study semi-structured interview designs (Matthews *et al.*, 2012; McCann *et al.*, 2013; Quirk and Haake, 2019). Using thematic analysis, Matthews *et al.* (Matthews *et al.*, 2012) and McCann *et al.* (McCann *et al.*, 2013) analysed the perspectives of program delivery staff, while Quirk and Haake (Quirk and Haake, 2019) focussed on experiences of the OAs as volunteers’ recruiters.

### Recruitment approaches

Recruitment approaches were mapped based on whether contact with the program participant was initiated or program initiated and by the potential reach of the recruitment strategy. The most frequently adopted approach to recruitment was purposive, with 4 papers (Chang *et al.*, 2009; Matthews *et al.*, 2012; McCann *et al.*, 2013; Quirk and Haake, 2019) relying solely on this approach. Clarke and Eves (Clarke and Eves, 1997) adopted the convenience approach to recruitment, while Bull and Milton (Bull and Milton, 2010), Coleman *et al.* (Coleman *et al.*, 1997), Kelly *et al.* (Kelly *et al.*, 2019), and Spencer *et al.* (Spencer *et al.*, 1998) adopted a mix of recruitment approaches. Bull and Milton (Bull and Milton, 2010) used a combination of purposive and convenience recruitment approaches; Spencer *et al.* (Spencer *et al.*, 1998) and Kelly *et al.* (Kelly *et al.*, 2019) included purposive, convenience and universal approaches, while Coleman *et al.* (Coleman *et al.*, 1997) built in purposive, convenience, random and universal approaches. Seven papers (Clarke and Eves, 1997; Coleman *et al.*, 1997; Spencer *et al.*, 1998; Chang *et al.*, 2009; Bull and Milton, 2010; Matthews *et al.*, 2012; Kelly *et al.*, 2019) sought to engage with potential participants directly and in a personal manner (active strategy), three of which complemented this approach with passive recruitment strategies such as public TV broadcasts, media and news coverage, and print materials(Coleman *et al.*, 1997; Spencer *et al.*, 1998; Kelly *et al.*, 2019).

See Table 2 summary of passive and active strategies and approaches adopted.

All quantitative papers reported recruitment rates (Clarke and Eves, 1997; Coleman *et al.*, 1997; Spencer *et al.*, 1998; Chang *et al.*, 2009; Bull and Milton, 2010; Kelly *et al.*, 2019), however, just Coleman *et al.* (Coleman *et al.*, 1997) and Spencer *et al.* (Spencer *et al.*, 1998) evaluated efficacy of recruitment strategies. Coleman *et al.* (Coleman *et al.*, 1997) described recruitment as the proportion of respondents who became active participants based on the reach of the recruitment strategies. Spencer *et al.* (Spencer *et al.*, 1998) defined recruitment as the proportion of eligible respondents who enrolled, the only study to appraise recruitment strategies based on cost per participant recruited. Bull and Milton (Bull and Milton, 2010) adopted two measures for reporting recruitment rates; those recruited opportunistically with an estimated number of consultations or ‘opportunities’ in a 12-week recruitment period used as the denominator for calculating recruitment rates. To calculate the recruitment rate for those invited via a disease register, the number of invitations sent out was the denominator, and the number of responses expressing interest as the numerator, however this rate does not represent the proportion of participants going on to commence the PA program following their initial expression of interest to the invitation (Bull and Milton, 2010). Chang *et al.* (Chang *et al.*, 2009) measured recruitment rate as the number of women providing information for screening as the denominator, and the numerator as the number completing screening. Clarke and Eves (Clarke and Eves, 1997) measured recruitment using the number of participants referred by GPs divided by the number of participants volunteering to participate, while Kelly *et al.* (Kelly *et al.*, 2019) presented recruitment rates as the proportion of participants recruited for each of the individual recruitment strategies.

### Other measures

Clarke and Eves (Clarke and Eves, 1997) analysed recruitment rates based on participants stage of change for PA [Transtheoretical Model (TTM)], and participant’s decisional balance, self-efficacy, and perceived barriers according to their stage of readiness (precontemplation, contemplation, or preparation). Chang *et al.* (Chang *et al.*, 2009) included measures of intervention efficacy in addition to recruitment measures. See Table 3 for a summary of intervention type, recruitment variable and measures reported in each study.

**Table 3:** Intervention type, recruitment variables and summary measures of included studies

Citation	Study type	Participants (*n*)	Female (%)	Mean age (years)	Intervention type	Reported recruitment variables	Main summary measures
Bull and Milton (2010)	Quant	526	57	54	PA prescription in primary care	Recruitment rates	% of approached patients who enrolled in study
Change *et al.* (2009)	Quant	129	100	26.4	Diet, physical activity, stress management program	Recruitment rates	% of people eligible after screening who enrolled in study
Clarke and Eves (1997)	Quant	393	69.5	45.13	PA prescription in primary care	Recruitment rates	% of patients referred by GP who enrolled in study
Coleman *et al.* (1997)	Quant	120	80	72	Senior centre-based health promotion trial with exercise component	Recruitment rates (total and by recruitment method)	% of respondents who enrolled in study; % of enrolees from each recruitment method
Spencer *et al.* (1998)	Quant	249	90	Not stated	Aquatic exercise program	Recruitment rates (total and by recruitment method); cost of recruitment by method	% of respondents who were eligible and enrolled in study; % of enrolees from each recruitment method; cost per enrolee by recruitment method
Kelly *et al.* (2019)	Quant	927	0	50.7	Community-based PA programme	Recruitment rates	% of respondents that registered for the programme
Matthews *et al.* (2012)	Qual	28	Not stated	Not stated	Walking groups	Type of recruitment (active/passive); perceptions of effective recruitment methods	Thematic analysis of responses
McCann *et al.* (2013)	Qual	25	Not stated	Not stated	Community health PA and healthy eating programs	Recruitment and retention strategies	Thematic analysis of responses
Quirk and Haake (2019)	Qual	13	Not stated	Not stated	Mass PA events—parkrun	Experiences of the use of Outreach Ambassadors as a recruitment strategy for those with Long Term Health Conditions into parkrun events	Thematic analysis of responses

### Recruitment outcomes

Spencer *et al.* (Spencer *et al.*, 1998) employed a suite of recruitment strategies to engage older people from Washington State in the USA into aquatic exercises programs. Recruitment strategies included Arthritis Foundation recruitment letters (30.5% of those enrolled came from this source), television coverage (16.1% of those enrolled), public service announcements (16.1%), Doctor referrals (12.1%), newspaper advertisements (9.6%), and other methods including word of mouth and flyers in targeted locations (13.8%). They screened 1018 people for eligibility and achieved a recruitment rate of 65% (Spencer *et al.*, 1998). Recruitment per participant from news articles came at a cost of $84, $47 for letters, and flyers were $42, costly options in comparison to free TV coverage, public service announcements and word of mouth (Spencer *et al.*, 1998).

Coleman *et al.* (Coleman *et al.*, 1997) reported on the recruitment of African American adults aged 55 and over for a senior centre-based health promotion program with a PA component in Seattle, USA. A recruitment rate of 46% was achieved, with ‘phonathons’ or ‘call-centre style’ recruitment the most successful strategy (accounting for 33% of those recruited). This was followed by printed materials—flyers and posters (33%), word of mouth (26%), and others (8%). Using community leaders of similar age and ethnicity to the target population for this ‘phonathons’ may have boosted recruitment numbers (Coleman *et al.*, 1997).

Chang *et al.* (Chang *et al.*, 2009) used peer recruiters aiming to engage low-income African American and White obese women into a 10-week PA program located in Michigan, USA. The strong emphasis on peer recruiters being culturally sensitive and using a personal approach to screening and inviting each participant resulted in 66.5% of eligible women enrolling in the PA program (Chang *et al.*, 2009).

Bull and Milton (Bull and Milton, 2010) used both opportunistic GP consultations and targeted recruitment letters from a disease register across three sites to recruit into a PA program delivered in a primary care setting in London, UK. Recruitment letters sent from a disease register bore varied results, with recruitment rates across the three sites ranging from 9 to 59%. The targeted letters were more effective than GP referrals conducted opportunistically (Bull and Milton, 2010).

Kelly *et al.* (Kelly *et al.*, 2019) used a combination of gender sensitive recruitment strategies to attract inactive men into a community-based PA program across Ireland. The authors calculated the proportion of participants recruited by each strategy used. Of the 927 participants, 31.2% were recruited using word of mouth and a further 23.3% cited newspaper or social media as their source of recruitment. While local service clubs accounted for 16.2% of recruited participants, local sports partnership for 10.3%, family and friends 8.4%, and health services 5.8% (Kelly *et al.*, 2019).

Clarke and Eves (Clarke and Eves, 1997) relied upon GP referrals and achieved a recruitment rate of 58.4% into a three-month prescription exercise program delivered across the UK. The majority of inactive participants recruited were in the contemplation (48.5%) or preparation (44.1%) stages of change for PA; suggesting an individual’s stage of readiness may influence the success of GP referrals as a recruitment strategy (Clarke and Eves, 1997). Without stage of change data reported for those who were invited to participate but not successfully recruited, it is difficult to draw conclusions (Clarke and Eves, 1997).

### Qualitative studies

Thirteen semi-structured interviews with OA for parkrun events across England, UK were reported in one included qualitative paper (Quirk and Haake, 2019). OA were parkrun volunteers with either a long term health condition (LTHC) lived experience or were caring for someone with a LTHC. OA’s engaged with the LTHC community acting as facilitators of recruitment and engagement in parkrun. Quirk and Haake (Quirk and Haake, 2019) do not report the recruitment rates achieved, however, thematic analysis revealed challenging perceptions of who participates in parkrun as essential for the successful recruitment of underrepresented participants living with LTHCs. LTHC groups experience different barriers to participation and without adequate policies, structures (such as courses suitable for those with low vision or using wheelchairs) and diverse communication strategies, recruitment of this hard-to-reach group will be sub-optimal (Quirk and Haake, 2019). Quirk and Haake (Quirk and Haake, 2019) findings emphasized the importance of engaging with stakeholder and advocacy groups to gain recognition of parkrun as a legitimate and inclusive PA opportunity (Quirk and Haake, 2019).

Matthews *et al.* (Matthews *et al.*, 2012) conducted semi-structured interviews and case studies with managers and project coordinators to explore recruitment strategies adopted during implementation of walking programs across the UK. Findings demonstrated choice of recruitment strategy/ies were driven by resources, program aims, skills and knowledge of practitioners (Matthews *et al.*, 2012). Programs with walking as the stated aim often had no targeted approach to recruitment and utilized passive strategies such as displaying promotional materials in local community spaces. Those with health aims or an identified target group adopted active, purposive recruitment strategies (word of mouth and relationship building with target organizations) (Matthews *et al.*, 2012). Matthews *et al.* (Matthews *et al.*, 2012) found programs using passive recruitment strategies attracted participants already physically active, while those with targeted active recruitment strategies recruited inactive participants—with word of mouth being the most effective (Matthews *et al.*, 2012). Passive recruitment methods such as advertising and newsletters, or a combination of both active and passive methods were more frequently adopted than active recruitment methods alone.

McCann *et al.* (McCann *et al.*, 2013), adopting a similar approach to Matthews *et al.* (Matthews *et al.*, 2012) conducted semi-structured interviews with those responsible for the delivery of organized PA programs in Australia; finding most program organizers made use of active recruitment strategies such as word of mouth (72%), links with key organizations and groups (64%), referrals (40%), cross promotion of programs (32%), and face to face contact through one off festival, presentations and vox pop surveys at shopping centres (20%); these were often used in conjunction with passive recruitment strategies—namely, printed materials (64%) and the printed news media (52%). There was consensus among program organizers that word of mouth was the most effective recruitment strategy for engaging with ethnic and minority populations (McCann *et al.*, 2013).

### Quality assessment

The assessment undertaken using AARQS (Foster *et al.*, 2011; Cooke and Jones, 2017) demonstrates the quality of reporting on recruitment for the included five quantitative papers in this review as ‘high’ with four out of the five studies achieving a four out of a possible five on this scale. Just 2 of 6 quantitative papers reported the efficacy of recruitment methods. No papers reported on the time spent planning recruitment, with just 4 papers reporting on the time spent implementing recruitment strategies. Authors 1 and 2 achieved 100% consistency when independently applying AARQS (Foster *et al.*, 2011; Cooke and Jones, 2017). Overall scores for each paper in Table 1.

## DISCUSSION

The purpose of this review was to examine strategies used to recruit adults into established, ongoing organized PA programs. Despite the importance of recruitment for program implementation, scale-up and sustainability, this review yielded just nine relevant studies. Among those, the measures of recruitment were reported inconsistently, and all papers failed to report on every item on the AARQS (Foster *et al.*, 2011; Cooke and Jones, 2017).

The variation in measures used to report recruitment rates may be related to the nature of the recruitment strategies adopted (passive or active), whether the strategy was purposive, convenience-based, or universal in scale, and whether a combination of strategies was used. Just one study reported on the reach of the recruitment strategies based on segmentation of the target audience (Clarke and Eves, 1997). This used the TTM, but because participants in the action and maintenance stages were excluded it was unclear if the recruitment strategy was likely to be more successful in reaching inactive or already active participants based on their stage of readiness. Kleis *et al.* (2021) recently reported in their review of PA interventions that variations in PA program efficacy can partly be explained by high proportions of participants recruited in the action and maintenance stage at baseline. While in an earlier study, using a range of recruitment strategies, the main reason for exclusion from the PA program was participants were already physically active (35% from all recruitment strategies) (Spencer *et al.*, 1998). Future studies focussing on measuring efficacy of recruitment strategies should consider including an assessment of the stage of readiness of participants recruited using various strategies, to provide insights into the extent to which these can successfully reach and engage individuals at varying levels of intention and participation in PA.

Building the capacity of practitioners to tailor recruitment strategies for the inactive segments of the population, particularly those experiencing barriers to PA and other forms of disadvantage, may contribute to increasing overall population PA. There is a growing body of evidence that mass PA events (Murphy *et al.*, 2015), organized PA opportunities that are universally available and community wide programs represent strong investments for increasing PA (Bellew*et al.*, 2020; Milton *et al.*, 2021). Yet, there are still gaps in the evidence as to how these various opportunities can effectively recruit inactive and hard-to reach populations (Carroll *et al.,* 2011).

Coleman *et al.* (Coleman *et al.*, 1997) provides some promising results for the use of active telephone follow-up (‘phonathons’) for recruitment into organized PA programs, however, the potential interrelationship between the various recruitment strategies adopted is unclear. It is difficult to ascertain if the recruitment levels reflected the success of the ‘phonathon’ strategy, or the result of participants being exposed to multiple recruitment strategies, thus supporting their readiness to participate when the phone call was received. While this study demonstrated some success with the use of a phonathon, in the modern environment this strategy may not be practical or yield similar results given the large proportion of the population without phones listed in public area-based phone books (Lavrakas, 2008).

Despite the ubiquitous use of social media platforms, just one paper mentioned the use of such platforms (Kelly *et al.*, 2019). Two qualitative studies (Matthews *et al.*, 2012; McCann *et al.*, 2013) highlighted the tendency of practitioners to rely on recruitment strategies familiar to them rather than seeking evidence-based options. There appears to be a strong reliance on strategies such as word of mouth, referrals, dissemination of print materials, cross promotion of programs and linking with local organizations (Priest *et al.*, 2008; McCann *et al.*, 2013).

The studies included in this review provided limited insights into the suitability of recruitment approaches for those experiencing barriers to PA or other impediments to reach and engagement, particularly those from Culturally and Linguistically Diverse populations, older people, or people from low socio-economic backgrounds (O’Driscoll *et al.*, 2014; Ball *et al.*, 2015; Pels and Kleinert, 2016). Nevertheless, all included studies noted successful recruitment is contingent upon ‘knowing your audience’ and shaping suites of recruitment strategies with acceptability to the demographic features of the participants that you are most interested in attracting to the PA program. The qualitative study conducted by Quirk and Haake (Quirk and Haake, 2019) explored recruitment to parkrun, indicated that understanding the lived experience of those who experience barriers to organized PA can assist in breaking down preconceived ideas and undertaking recruitment in ways that are acceptable and practical.

### Limitations

The search strategy may have omitted articles relevant for inclusion. Due to the heterogeneous nature of the recruitment strategies and outcomes measures, it was not possible to conduct a meta-analysis or a structured assessment of publication bias. Due to the low number of publications included in this review an analysis of recruitment strategies based on adult life stage or age group stratification was not possible and the findings may not be transferable to recruitment of children or young people into organized PA. This review includes publications both pre (prior to March 2019) and post (post March 2019-) COVID pandemic, social distancing restrictions had a profound impact on the PA sector and thus on recruitment strategies available within the context of social distancing.

## CONCLUSION

This review found culturally sensitive, gender sensitive and socially inclusive recruitment strategies based on building personal relationships show promise for engaging hard-to-reach populations, while demonstrating inconsistencies in the way recruitment into established organized PA programs are reported in the peer reviewed literature. The adoption of consistent reporting standards and measures for recruitment strategies into PA programs will provide program planners and implementers with a better understanding of the potential efficacy of different recruitment methods. To improve the consistency and standardization of reporting the adoption of Foster *et al.*’s (Foster *et al.*, 2011) ARRQS with Cooke and Jones’s (Cooke and Jones, 2017) adaptions is recommended to be included in published PA program evaluations. Additionally, where possible, practitioners and evaluators should measure recruitment rates in relation to the size and composition of target populations, stage of readiness for PA and report recruitment processes followed to enable replication and adaption in different contexts. This is especially important for targeting hard-to reach segments of the population that may experience barriers to PA and other social inequities. Building the evidence base concerning recruitment into PA programs is essential for improving effectiveness, tackling inequities in PA participation, and making efficient use of limited community resources.

## Supplementary Material

Supplementary material is available at *Health Promotion International* online.

Supplementary File 1. Search concepts and terms.

Supplementary File 2. The Assessment of Recruitment Reporting Quality Scale (ARRQS) (Foster *et al.*, 2011; Cooke and Jones, 2017).

Supplementary File 3. Revised recruitment strategies definitions from Foster *et al.* (Foster *et al.*, 2011).

daad050_suppl_Supplementary_Material_S1

daad050_suppl_Supplementary_Material_S2

daad050_suppl_Supplementary_Material_S3
