# Supplementary material for: Engaging adults in organized physical activity: a scoping review of recruitment strategies
Source: Health Promot Int. 2023 May 26;38(3):daad050. doi: 10.1093/heapro/daad050 (PMC10214989; doi:10.1093/heapro/daad050)
Supplement: daad050_suppl_Supplementary_Material_S1 [file daad050_suppl_supplementary_material_s1.docx]

**Supplementary File 1: Search concepts and terms**

| **Physical activity** | **Recruitment methods** | **Participation** |
| --- | --- | --- |
| Physical* active* OR exercise OR physical* fit* OR physical* endurance OR physical* exert* OR sport* OR motor OR recreation* activity OR aerobic active* OR organised sport OR health club OR fitness centre OR gym OR recreation centre OR physical training OR sport training OR sport* club OR pool OR community cent* OR sport* facility OR sport* cent* OR leisure cent* | Recruit* intervention* OR engage* method* OR community engagement OR social marketing recruit* OR marketing OR sport market* OR incentive* OR recruit* strategy* OR relationship management OR persuasion OR reward OR reinforce* | Consumer participate* OR attendance OR adopt* OR presence OR taking part OR engage* OR play* OR partake OR evaluation OR case study OR intervention OR intervention trial |
