# Supplementary material for: Engaging adults in organized physical activity: a scoping review of recruitment strategies
Source: Health Promot Int. 2023 May 26;38(3):daad050. doi: 10.1093/heapro/daad050 (PMC10214989; doi:10.1093/heapro/daad050)
Supplement: daad050_suppl_Supplementary_Material_S2 [file daad050_suppl_supplementary_material_s2.docx]

**Supplementary File 2: The Assessment of Recruitment Reporting Quality Scale (ARRQS)** (**Cooke and Jones, 2017; Foster et al., 2011)**

On this scale, one point is given for each of the items (listed below) the paper explicitly or adequately described, with 0 is given for each item failing to be reported.

1. Did the paper report where the population was recruited?

2. Did the paper report who conducted the recruitment?

3. Did the paper report the time spent planning recruitment?

4. Did the paper report the time spent conducting the recruitment?

5. Did the paper report recruitment results for the target population?

Studies scoring 3 or less were ‘low quality’, those scoring between 4 and 5 were considered ‘high quality’ (Cooke and Jones, 2017; Foster et al., 2011).
