# Supplementary material for: Engaging adults in organized physical activity: a scoping review of recruitment strategies
Source: Health Promot Int. 2023 May 26;38(3):daad050. doi: 10.1093/heapro/daad050 (PMC10214989; doi:10.1093/heapro/daad050)
Supplement: daad050_suppl_Supplementary_Material_S3 [file daad050_suppl_supplementary_material_s3.docx]

**Supplementary File 3: Revised recruitment strategies definitions from Foster et al. (2011)**

Recruitment strategies were classified as ‘active recruitment strategies’, ‘passive recruitment strategies’ or both, in accordance with the definitions below.

*Active recruitment approaches*: a recruitment strategy that requires those implementing a PA project/program or intervention to make the first contact with a potential participant (for example, phone calls, face to face invitations, word of mouth and referrals).

*Passive recruitment approaches*: A recruitment strategy that requires a potential participant to make the first contact with those implementing a PA program/project or intervention (for example posters, flyers, newspaper advertisements, media and news pieces).
